# Cultural adaptation of ‘Healthy Dads, Healthy Kids’ for Hispanic families: applying the ecological validity model

**DOI:** 10.1186/s12966-020-00949-0

**Published:** 2020-04-21

**Authors:** Teresia M. O’Connor, Oriana Perez, Alicia Beltran, Isabel Colón García, Elva Arredondo, Ruben Parra Cardona, Natasha Cabrera, Debbe Thompson, Tom Baranowski, Philip J. Morgan

**Affiliations:** 1 USDA/ARS Children’s Nutrition Research Center, Baylor College of Medicine, 1100 Bates Street, Houston, TX 77030 USA; 2 School of Public Health, San Diego State University, San Diego, CA USA; 3 Steve Hicks School of Social Work, University of Texas at Austin, Austin, TX USA; 4 Dept of Human Development and Quantitative Methodology, University of Maryland, College Park, MD USA; 5 Priority Research Centre for Physical Activity & Nutrition, Faculty of Education & Arts, University of Newcastle, Newcastle, Australia

## Abstract

**Background:**

Healthy Dads Healthy Kids (HDHK) is a unique lifestyle obesity intervention for fathers and children that demonstrated weight loss among the fathers and behavior change among fathers and children in Australia. The program is gender-tailored to specifically target fathers for weight loss and 5–12 year old children for obesity prevention. The aim of this formative study was to examine an Expert Panel’s and Hispanic Family Panel’s perceptions about the program and suggestions for the cultural adaptation of HDHK for Hispanic families in southwestern US.

**Methods:**

Forty-four Hispanic participants (22 fathers, 13 mothers and 9 children) made up the Family Panel. They participated in 1–5 study contacts (focus groups, online survey, and/or interviews). The scripts and qualitative guides assessed participants’ perceptions of the HDHK content and material using the Ecological Validity Model. Studies were conducted in English or Spanish, depending on the preference of the participant. Focus groups and interviews were audio-recorded, transcribed, translated, and thematically coded. Findings were reviewed with the Expert Panel who helped inform the cultural adaptation.

**Results:**

80% of parents were foreign-born, 57% spoke only Spanish at home, and 60% did not graduate from high school. Several themes emerged to inform the cultural adaptation of the program. Parents agreed with the HDHK goals and recommended the program place greater emphasis on parenting and limiting children’s screen time. Some mothers and fathers wanted greater mother engagement. Weekly videos and a Facebook group emerged as favorite alternative options to engage mothers. Greater promotion of *familism* (inclusion and impact on whole family) was recommended for the program goals and activities. Gender roles for mothers and fathers, and differences in how fathers interact with male and female children, emerged and should be considered in program activities. Several barriers to father engagement surfaced, including lack of time due to work schedules, physically demanding jobs, concerns of caring for children without mother, fathers’ current fitness/weight, and lack of knowledge of how to eat more healthfully. The reading level of the HDHK materials was too high for some parents.

**Conclusion:**

Findings from these formative qualitative studies informed the cultural adaptation of HDHK for Hispanic families, to account for literacy level, cultural values, and barriers to participation and engagement.

## Introduction

Obesity remains a public health problem in the US. As a group, Hispanic children are at higher risk for overweight and obesity compared to Non-Hispanic white children [1], putting them at higher risk for obesity-associated medical conditions [2]. On average, Hispanic men are also at greater risk for obesity compared to other men in the US [3] highlighting a health disparity for Hispanic families. Prior research supports the important influence fathers have on their children’s eating and activity behaviors [4–6] and data are accumulating that Hispanic fathers are an important influence in their children’s lifestyle behaviors [7–9]. However, recent systematic reviews of pediatric obesity treatment and prevention trials have reported fathers have been only minimally engaged or targeted by such programs [10, 11], despite their influential role on the health behaviors and outcomes of youth.

Healthy Dads, Healthy Kids (HDHK) is an evidence based, healthy lifestyle program developed in Australia to promote healthy eating, physical activity (PA), and weight loss for overweight or obese fathers, as well as healthy lifestyle behaviors among their 5–12 year old children for obesity prevention [12, 13]. The 9 week program, based on social cognitive [14] and family systems theories [15], was the first to successfully target fathers in a family-oriented lifestyle program. The program helped fathers reach their personal weight loss goal and positively influence their child’s eating and PA behaviors. HDHK has been evaluated in randomized controlled efficacy [12], effectiveness [13] and dissemination trials [16] in Australia. All trials demonstrated significant and clinically meaningful effects on fathers’ weight loss, and fathers’ and children’s lifestyle behaviors [12, 13, 16–18]. HDHK primarily targets fathers and children, but includes mothers and siblings in one session to reach the entire family. Culturally adapting HDHK for Hispanic families offers promise as a novel approach to promote weight loss for Hispanic men and obesity prevention for Hispanic youth to reduce health disparities.

The goal of culturally adapting HDHK is to enhance engagement of low-income Hispanic fathers and families, and maximize the program impact on Hispanic families [19, 20]. Drawing from the mental health field, it has been found that programs that have multiple adaptations for a specific cultural group are more effective than those that only have a few adaptations [21, 22]. Cultural adaptation includes systematically enhancing the language and cultural content of the program, while accounting for the context of the new target group to improve recruitment and retention of the target population, and promote desired parent and child outcomes [19, 20]. For example, a qualitative study with Hispanic parents (mothers and fathers) in the US illustrated they desired to participate in parenting interventions that are culturally relevant to them, promoted collaboration and respect, included the cultural values of *respeto* and *familism*, facilitated learning in group settings, and promoted a sense of community [23]. Cultural adaptation may therefore promote satisfaction and relevance for the target population, but the adaptation needs to retain the core intervention components that promoted efficacy in original trials of the program [21].

The Ecological Validity Model (EVM) proposed by Bernal and Sáez-Santiago is a framework to guide the culturally sensitive adaptation of interventions for new cultural groups [21]. EVM recommends adaptations across eight dimensions to culturally center an existing intervention: program goals, concepts, methods, content, persons, metaphors, language, and context. Table 1 provides definitions for the eight dimensions. The aim of this study was to culturally adapt HDHK for Hispanic families in the US based on the EVM.

**Table 1:** The dimensions to consider for culturally centering a program in the Ecological Validity Model (EVM)

Culturally Centering Elements	Bernal and Sáez-Santiago’s EVM definition^**a**^
Goals	Agreement between the intervention’s intended goals and participant’s understanding of the goals of the program. Consideration should be made of participants’ values, customs, and traditions.
Concepts	How theoretical constructs of the program are conceptualized and communicated to participants.
Methods	The procedures and activities to follow for the achievement of the program goals.
Content	The values, customs, and traditions held by a cultural group to be considered when delivering and assessing a program.
Persons	The cultural understanding of the participant-facilitator relationship in a program.
Metaphors	The cultural understanding of certain symbols, sayings and concepts that could affect recruitment and engagement in the program.
Language	A mechanical translation of the program with consideration of the dialect and word choice by country of origin and current living environment. The emotional expression of language and mannerisms should also be considered.
Context	The overarching socio-economic background of the participant, social support and relationship to their culture of origin.

^**a**^Dimension descriptions were adapted from [21]

## Methods

The cultural adaptation involved two sets of contributors (i) a panel of Hispanic fathers, mothers and children (Family Panel) and (ii) a panel of researchers (Expert Panel). The Family Panel included Hispanic parents and some of their children who participated in a formative qualitative study with multiple study contacts to help inform the cultural adaptation of HDHK described in this manuscript. In addition, the Expert Panel (coauthors EA, RPC, NC, PM), which included the original developer of Healthy Dads Healthy Kids (PM), helped guide the cultural adaptation of the HDHK program.

### Participants

In order to reach a similar sample as the planned feasibility trial, recruitment for the Family Panel occurred at two Texas Children’s Health Plan (TCHP) Center for Children and Women clinics in Houston, TX. TCHP is a Medicaid and Children’s Health Insurance Plan (CHIP) provider for qualifying, low-income children and women in the southeastern part of Texas. Participants were recruited by posting and handing out fliers about the study in the waiting rooms of the two clinics. The same method was used to recruit mothers and fathers, but fathers were often asked to contact the study staff about the study by their partner (Mother) who received a flier in clinic. Children were recruited from the parents who were part of the Family Panel. To qualify, parents had to self-identify as Latino or Hispanic, be able to read or write in Spanish and/or English, and be a mother or father-figure (i.e., significant male figure in the child’s life who participates in raising the child) of a 5–11 year old child. Their child also had to be a TCHP member and a patient at one of the TCHP Center for Children and Women clinics.” Father figures” included self-identified biological fathers, stepfathers, older adult brothers, grandfathers, or uncles. The person identifying as a father-figure will be referred to as ‘father’ in this study. Participants were excluded if the child or parent had a disease affecting their dietary intake (severe GI disease or food allergies), PA behaviors (e.g., physical disability, severe asthma), cognitive functioning (e.g., Down’s syndromes, autistic spectrum disorder), or psychiatric functioning (e.g. schizophrenia) similar to what was planned for the feasibility study because these conditions could affect their ability to take part in a program like HDHK. Participants were also excluded if they had plans of moving away from the area in the next year, which would prevent them from continuing participation. Participants were invited to participate in as many study activities as they wanted, with a range of one activity (*n* = 11 parents, *n* = 9 children) to five activities (n = 1 parent). During the course of the study, an on-going rolling recruitment for the parent panel was conducted to replace those parents that could not or did not want to participate further, in order to ensure the panel was large enough to provide diverse perspectives and input. Parents were paid $30 for each activity they took part in, for no more than five activities. Children received $20 for taking part in one focus group.

### Overview of the HDHK program

The original HDHK [12, 13, 24] was a nine-week, group-based program for overweight or obese fathers and their elementary-school aged children. The program participants met weekly for 90-min with three distinct parts, a) welcome/check-in, b) separate education components for fathers and children, and c) combined PA component for fathers and children. Each week covered different topics for the fathers and children. The PA component included facilitator-guided activities in rough and tumble play, fundamental movement skills, and fun fitness. All the physical activities encouraged fathers and children to take part together in fun, challenging games that they can also do at home. Fathers and children attended all the sessions together, and mothers and siblings were invited to take part in one session.

The objective of cultural adaptation is to augment the ecological and external validity of the program for a different cultural group than was originally intended. However, as described in the EVM it is important to retain the core components in the program to increase the chances it will be effective for a new target population [21]. Several core components of HDHK were identified to be retained [12, 13]. Targeting fathers in a gender-tailored manner was the fundamental premise of HDHK and therefore a core component [25]. Second, fathers were encouraged to reciprocally change their lifestyle behaviors and to be good role models for their children, while children were encouraged to support their father in weight loss efforts by prompting him at home to be more active and eat more healthily (termed *reciprocal reinforcement* [25]*)*. Third, fathers were taught authoritative parenting techniques and encouraged to spend more quality time with their child. Fourth, the program was delivered to a group with an emphasis on engaging in PA and healthy eating habits together as a family. These core components helped achieve the primary aims of the original HDHK program, which were weight loss for fathers and obesity prevention for children.

### Procedures

Parents from the family panel were invited to participate in multiple study activities (Table 2), over a 12-month period (6/2017–6/2018). The research team held their first call with the Expert Panel in May 2017 to discuss the cultural adaptation of HDHK. This discussion informed the development of the focus group guide for the first contact with parents from the Family Panel. The focus groups with the parents introduced the HDHK program to the fathers and mothers in English or Spanish and allowed them to provide their initial impressions of the program. The focus group guide further explored parents’ thoughts of the HDHK goals and on several HDHK concepts, methods, content, and language translation issues. The Expert Panel was presented the initial findings from the focus group on a teleconference in the fall of 2017. During the call, the panel team provided input on the proposed program names, logos, and session formats. The discussions and questions that arose during this review of the focus group findings and initial plans for cultural adaption, informed the questions for the online-survey and follow-up in-depth interview. The online survey provided a format for the parents to provide quick responses for several closed ended questions, such as preferences for layouts, logos, colors, and names. The follow-up interviews explored the reasons behind their selections, as well as barriers and facilitators for participation and engagement, and additional resources they might find helpful. Asking about this in individual interviews allowed discussion of more sensitive topics that participants may not have wanted to bring up during the focus group discussion. The findings from the first three study activities were summarized to the Expert Panel during a two-day, in-person meeting in the spring of 2018. With this information, the Expert Panel helped the research team prioritize and decide on how to best adapt the program materials and content to address the needs and preferences of the Hispanic community. The discussions during the in-person meeting, identified a few additional areas the Expert Panel recommended the research team explore with the Family Panel. These included questions about fathers’ motivations to participate in the program, thoughts about their own health and specific concepts discussed in the program (e.g. “changing the shape” of their work week to be able to spend more time with their child (ren)). This informed the next set of interviews with the fathers. Additionally, the research team and Expert Panel decided to keep the focus on fathers during the program, but thought it would be important to identify ways to engage mothers more in the culturally adapted version of HDHK. This informed the last set of interviews with the mothers. These last study activities, as well as the focus group with children, allowed the research team to make final decisions about the cultural adaption of the program to be assessed in a feasibility trial. Participating fathers were also invited to take part in a cognitive interview to inform the Spanish translation and adaptation of a survey instrument to assess fathers’ parenting practices regarding PA and eating for children. Information from the cognitive interview will not be summarized here, other than fathers’ feedback on language. Table 2 provides an overview of the iterative studies and EVM dimensions addressed at each step.

**Table 2:** Study Contacts with Family Panel

Type of Contact	Purpose	Ecological Validity Model dimension explored	n
Focus Groups (three) 1 Spanish-speaking, Fathers and Mothers 1 English-speaking, Fathers and Mothers 1 Spanish-speaking, only Fathers	Identify whether key components of the HDHK need to be adapted or changed for the Houston Hispanic community. Identify any new concepts that may need be added to HDHK to meet the needs of Hispanic families in Houston.	Goals Concepts Methods Content Language	17 (10 Fathers, 7 Mothers)
Online Survey, Fathers and Mothers	Introduce and get feedback on parental preferences for the initial ideas for adaptation of program name, logos and colors; and the initial adaptation of play cards.	Content Metaphors Language	21 (11 Fathers, 10 Mothers)
Follow-up Interviews, Fathers and Mothers	Clarify responses from online survey on program colors, adapted name and logos and initial adaptation of play cards. Assess barriers and facilitators for having fathers and their children a) be more active together, b) eat healthier, and c) participate in the HDHK program. Identify preferences and suggestions for additional resources that will be provided during the implementation of the program.	Methods Content Metaphors Language Context	16 (7 Fathers, 9 Mothers)
Cognitive Interviews, only Fathers	Assess their understanding of instructions and items and ease in completing a survey questionnaire regarding their parenting practices. Assess whether the survey accurately reflects parenting practices they use to influence their children’s nutrition and PA behaviors and if additional concepts should be added.	Language Context Content	12 (All Fathers)
Interviews, Fathers	Assess fathers’ perceptions and goals of their own health, motivation to participate in a program like HDHK, reaction to program concepts (such as “changing the shape of the work week”), and understanding of terms and phrases used in the adapted material	Goals Concepts Methods Content Persons Language	5 (All Fathers)
Interviews, Mothers	Assess how mothers preferred to receive information and be engaged in the program when program sessions remained targeting fathers and children (a core component)	Goals Concepts Methods Content	7 (All Mothers)
Focus Groups, Children 1 English-speaking 1 Spanish-speaking	Identify whether key components of the HDHK children’s materials and activities need to be adapted or changed for Hispanic children. Identify any new concepts that may need be added to HDHK children’s materials to meet the needs of Hispanic children.	Concepts Methods Content Context Language	9

In the first panel activity, participants received an overview of the original HDHK program through a short video, power point presentation, and/or review of physical copies of the original or partially adapted HDHK material (e.g. translated into Spanish for the Spanish speaking groups/interviews). Focus group and interview guides were developed to assess participants’ thoughts and input on the program with a focus on the eight dimensions of EVM [21]. For example, focus group participants were presented with the goals of the original HDHK program and the participants’ beliefs and desired outcomes of such a program were examined. In addition, the fathers’ thoughts on the weight management goal were further explored in interviews. A second example from the interviews, mothers were asked their preference from four options for how to best keep them engaged and informed about the program: a) a handbook given at the start of the program, b) weekly newsletter mailed to their house, c) weekly emails and/or d) short videos sent via Facebook group or text. An example of each was presented to them during the interview to examine and comment on.

Bilingual staff trained in qualitative methods conducted the focus groups and interviews. Panel activities were conducted in English or Spanish. Scripted open-ended questions were posed, followed by probes and/or prompts to clarify and expand on the responses when needed. All the focus groups and interviews were audio-recorded, professionally transcribed and, if necessary, professionally translated into English.

### Analysis

Bilingual staff reviewed all the transcripts and compared them to the original recordings for quality assurance of transcriptions and translations. English-language transcripts were coded using NVivo 11 (QSR International; 2015) independently by two out of four bilingual staff, who referred back to Spanish transcripts if needed for clarification. An unstructured thematic coding approach was used for each activity to allow codes to capture the voice of the participants. Codebooks were developed and maintained for each activity. Coding differences between coders were reviewed and consensus achieved through discussion. Reports summarizing the main themes and subthemes were developed for each research activity. Overarching and sub-themes were next identified across the reports of all the research activities, organized by the eight EVM dimensions. Illustrative quotes are provided at the end of some themes. Descriptors of the participant who provided a quote included parent or child, sex of the participant, the type of research contact the quote was derived from, and the language of the interaction. The study was reviewed and approved by the Institutional Review Board at Baylor College of Medicine (protocol 38237) and all participants provided signed informed consent and/or assent to participate.

## Results

A total of 44 people took part in the Family Panel, 22 fathers, 13 mothers and 9 children (6 boys and 3 girls). The mean age for the parents was 37.3 years (SD 8.4 years) and the mean age of the children was 9.2 years (SD 1.3 years). See Table 3 for full demographic descriptors. Participants took part in 1–5 of the study activities. The results of the qualitative analyses across all five activities are summarized below, organized by the EVM dimensions.

**Table 3:** Socio-demographics of parent and child panel

Demographic Variable	Number	Percent
Parent sex, *n* = 35		
Male	22	63**%**
Female	13	37**%**
Child sex, n = 9		
Male	6	67**%**
Female	3	33**%**
Parent education		
Less than High School	21	60**%**
High School/GED	10	29**%**
Greater than High School	4	11**%**
Family income		
< than $20,000	10	29**%**
$20,000- $39,999	22	63**%**
> $40,000	3	9**%**
Language spoken at home		
English	2	6**%**
Spanish	20	57**%**
Both English and Spanish	13	37**%**
Parent born in the US		
Yes	7	20**%**
No	28	80**%**
Country where born, if outside US		
Mexico	13	46**%**
Honduras	9	32**%**
Puerto Rico ^a^	1	4**%**
Colombia	1	4**%**
Guatemala	1	4**%**
Missing	3	11**%**
Family Country of Origin		
Mexico	16	46%
Honduras	8	23%
Colombia	1	3%
Guatemala	1	3%
USA	4	11%
Multiple - Other	4	11%
Missing	1	3%
Relationship with the child		
Father	18	51%
Step Father	3	9%
Other male relative	1	3%
Mother	13	37%
	**Mean**	**SD**
Parents Age (years) n = 35	37.31	8.4
Child Age (years) n = 9	9.22	1.3

The % values in some categories do not sum to 100% due to rounding

*GED* general education diploma

*US* United States

^a^Participant reported being born outside of US and identified country as Puerto Rico. Given the cultural difference of Puerto Rico from Latin American and South American countries, this was retained despite Puerto Rico being a territory of the US

### EVM dimension: goals

The parents in the Family Panel were presented with the six main goals of the HDHK program: 1) Teach fathers how their attitudes towards eating and PA influence the whole family; 2) Encourage fathers to be healthy, positive role models for their family; 3) Teach fathers effective ways to encourage healthy behaviors in their children; 4) Assist to prevent or manage obesity in children; 5) Help fathers achieve a healthy weight; and 6) Improve the relationship(s) between fathers and their child (ren) through PA and healthy eating. Several main themes emerged across the study activities regarding the HDHK goals.

*There is a need for a program that focuses on Hispanic fathers*. Subthemes included, the program would benefit Hispanic fathers’ and children’s health, improve bonding between father and child (ren), and make children happy. It was noted that there are other family based programs, but none specifically for fathers. Focusing on fathers was seen as unique and important because fathers are often overlooked and not always allowed to or interested in taking an active role in childrearing within Hispanic families.

*“Yes try to focus on making dads aware to actively participate in his family duty, because he is an important factor in the process of raising children, not only by being an example but in roles of recreation and spending time with their children alone without the mom being present. It’s not only moms. Like he said, they look at me like I’m an ogre. And women sometimes are to blame for that.”* (Father, Focus Group, Spanish)

*Facilitating father-child bonding was important.* Increasing bonding between father and child (ren) emerged as the goal parents liked most about the program. Subthemes included the program would allow fathers to get closer to their children, and it would allow them to spend more time with children doing fun activities. Participants believed both fathers and children would like this aspect of the program.

“[My daughter] *would be very happy that her dad is with her, participating*.” (Mother, Interview, Spanish)

*Hispanic men wanted to learn how to be healthy.* Fathers wanted to learn more about nutrition and how to be healthy for the benefit of themselves and to help their children be healthy. Fathers wanted to be healthy to continue to be present for their children and they wanted to help their children to learn about eating healthy and PA at an early age to benefit them later in life.

*“ … if you don’t take care of yourself, you’re not going to be there for your kids or your grand kids.”* (Father, Interview, English)

*Weight management for Hispanic men was important*. Of the fathers who participated in the interviews assessing fathers’ attitudes about their health, all agreed that weight management was important for fathers. Subthemes included that being overweight made fathers feel uncomfortable or poorly; and weight control was difficult. Reasons provided for the difficulty in weight control included ready access to fast food, lack of time to cook, ease of over-eating, and long work hours.

*“I believe they would think [having a healthy weight] is good …*. *.Because the majority of people want to live well.” (*Father, interview, Spanish)

However, one participant thought some other Hispanic men might feel offended by the program targeting weight management for men.

*Parents wanted the program to focus more on how to reduce screen time for their children and parenting skills for fathers*. Excess screen use by children was identified as a common problem that parents did not feel equipped to handle. Wanting to learn more about parenting in general and specifically for restricting screen use reoccurred as subthemes.

“*You need to understand that, as a parent, we don’t know what we’re doing wrong or what’s going on, you know? So we’re trying to learn ourselves to- what can be the best way to raise these kids because they don’t come with a manual*.” (Mother, focus group, English)

### EVM dimension: concepts

Four concepts from HDHK’s core components were explored across the study activities with parents. The core components included a) the focus on fathers, b) how men’s attitudes toward eating and PA influence the rest of the family, c) fathers are important role models for children, and d) reciprocal reinforcement.

- *The focus on fathers in the program by gender tailoring it to proactively engage fathers in their children’s eating and PA behaviors*: Four themes emerged regarding the focus on fathers. One theme was appreciation. Parents noted fathers were often overlooked and not given the opportunity to work on their relationship with their child. Many fathers and mothers appreciated a program specifically for fathers.

“ … *we almost never have programs for dads … The foundation of family here is the mother. The father is nonexistent*.” (*Father, focus group, Spanish*)

A few thought a father-only program may make it more likely for fathers to participate.

“ *maybe knowing that it’s going to be only dads, men … maybe that will encourage them more, that there won’t be moms, … they are not going to be the only ones*” (Mother, interview, Spanish).

Some mentioned the traditional role of Hispanic men not being as involved with their children may make some fathers reluctant to take part. A few recommended the focus should be on the whole family. In fact, the cultural concept of *familism* emerged several times when discussing who should attend the program with a few participants wanting mothers to take part in all aspects of the program.

*“No I think it would be good for moms to be included for all of it. To coexist as a family.”* Followed by “*Everyone. The whole family”* (Father followed by Mother, couple, Focus group, Spanish).

b. *Men’s attitudes toward eating and PA and how these behaviors influence the family*: Several themes emerged for this core component. Hispanic men may not be considered completely healthy. The reasons included a lack of exercise, having a ‘belly’, being overweight or in the process of losing weight, getting very tired while working, or eating a lot of fast food. Hispanic food preferences were identified as a barrier to adopting a healthier diet.

*“You know they will be like, no more tortillas and – I don’t know it is just I like to – I like Hispanic food, I like Mexican food. Part will be – yeah the food itself like – healthy food doesn’t taste good.”* (Father, interview, English)

Some Hispanic men desired to learn about eating more healthfully.

*“I believe they would take it into consideration, telling them about food that is healthier and cheaper, and teach them all the food that we eat … we are in a country that if you go to any corner you eat mostly bread, soda … that’s the only thing they’re going to eat … nowadays, energy– they say there is a lot of liquids that have energy … we don’t really see all the calories and sugar it contains.”* (Father, interview, Spanish).

Some Hispanic fathers did not believe other Hispanic fathers have positive attitudes about PA. This primarily related to Hispanic men being tired after physically demanding jobs. Having fathers and children be active together was perceived as one of the best parts of the program. Subthemes that emerged included fathers’ desire to know how to do exercises and find places to exercise with their children.

“ … *talking about the program, if you know that there is a program where you can be active with your children … I believe that is what everybody wants, to be active with the children*” (Mother, interview, Spanish).

c. *Fathers as important role models for their children*: Role modeling by fathers was viewed as important and a potential way to motivate men to participate in a program like HDHK. One participant expressed that children would be more likely to take part in the Fun Fitness component if they saw their father doing it.

“… *He wants to do it to be like dad* …” (Father, focus group, English)

d. *Reciprocal Reinforcement:* The themes that emerged for reciprocal reinforcement demonstrated that this core component was not liked by all. Some fathers expressed incredulity that their children would try to make them be healthier and it is usually parents who try to get their children to be more physically active and eat healthier.

*“I’m more active than they are”* (Father, focus group, English)

*“That our kids are helping us? In my particular case, it’s the other way around. … In my particular case, I don’t see my kid … How should I say it? Wanting to eat healthy. To them, it’s only pizza and pasta.”* (Father, focus group, Spanish)

Some participants noted that other parents might not respond well to their children’s attempts to help them be healthier

*“Don’t tell me what to do.” "Some fathers do, do that …* "(Father followed by Mother, focus group. English)

On the other hand, a few parents agreed they would have a positive response to their children’s attempt to help them be healthier.

*“Yes because sometimes I get home and my son ask me to go to the park for a bike ride, so he is who … So I say: 'let me rest a bit and we’ll go in a moment'”.* (Father. Focus group, Spanish)

A few children believed their father would accept coaching from them.

*“That is good to help him be healthy*” (Boy, focus group, Spanish)

However, most children expressed the sentiment that their father would not trust or listen to them if they told him what to do.

“*Well maybe not that good because you can’t actually boss him around*” (Boy, focus group, English)

### EVM dimension: methods

We explored five main HDHK methods: a) session schedules, b) activities, c) home games and sport activities, d) behavior change techniques, and e) how to engage mothers.

- *Schedule of the sessions*: The primary theme that emerged was the need to schedule sessions to accommodate busy work schedules for fathers. Weekday evenings or weekend mornings or afternoons were identified as best times to schedule sessions.
- *Group Session Activities*: Several themes emerged regarding the session activities. The parents suggested the program should avoid making participants feel they are being lectured. Subthemes included to cut down on time spent on lectures, increase playtime, make slides easy to understand, and avoid a lot of text. Some reported it is important to deliver health content to participants, but not to deliver too much information or material at once.

*“Maybe, it’s not that we are not going to get it, we are going to get it. But since it will be a lot of information, there won’t be so much enthusiasm in that. We have to enter little by little, like one step at a time, so that they take it into consideration.”* (Mother, interview, Spanish)

The PA components were well received. Many subthemes emerged including PAs that create a competitive environment for children and fathers would be motivating for them. Parents liked the idea of teaching basic sports skills on how to properly throw, run, jump, kick, etc. to children because some children grow up never learning this. Parents liked fun fitness because it offered a *“distraction”* for their children from video games and phones. Fathers mentioned they already did **“**wrestling-like” games with their children (rough and tumble play), and many who were not already playing such games were willing to try rough and tumble games with their children. Silly wrestling games with their children (rough and tumble games) may not be well received by some Hispanic men due to cultural beliefs about gender roles for fathers (see further discussion on machismo in Content section). The fitness level of fathers and children was identified as a concern for some, who felt the level of difficulty of some of the PA activities and fitness level required to perform the activities was high. They suggested variations or modifications may be needed for some. Overall, the children were excited to play rough and tumble games with their father. The children expressed their excitement for the games and thought this form of play would be fun and funny to play with their fathers.

“… *we get to play with our dad finally*.” (Boy, focus group, English)

Some children had concerns with playing PA games with their father because fathers would not have enough time to play with them due to work obligations, fathers would be too rough during playtime, fathers are not “playful,” and a few believed their father would dislike playing the games with them.

“*He never watches, he never sits down or watches the TV or lays down on the bed. He’s always outside working so he never has time to come inside and spend time with us*” (Boy, focus group, English)

Some parents echoed the idea Hispanic fathers are not that playful.

“*They don’t get involved in the sense that when it’s time to play, the one who plays and jumps with the children, in a lot of homes, is the mom. The father has the concept of being the provider in the home, so he came tired. So, maybe with this program, the dad will wake up … and he will know that this will help … both for their children and for them*.” (Mother, interview, Spanish)

c. *Home games and sport activities*: HDHK encouraged fathers and children to be active during the week by using a set of game cards to get them active at home. The game cards reinforced 15 fundamental movement and sports skills for children (movement, ball skills, balance and rough and tumble play). Each card illustrated the correct form and steps of each skill and provided games for the father and child to play at home to practice the skill. The themes that emerged for participants’ reaction to the game cards, included that the cards gave families ideas of activities they can do to increase their PA. Parents stated they would like to try many of the games with their child. They offered variations for some of the games to illustrate how they remembered playing a similar game as a child. They also provided names in Spanish or English for what they called some of the games as a child. Some suggested to make the instructions easier to read and cards more colorful. Children recommended the reading level should be lower for their parent to read the rules of the games. In fact, the overall observation during the focus groups, interviews and cognitive interviews was some fathers’ reading level appeared to be lower than the seventh grade level.
d. *Behavior Change Techniques*: In addition to the game cards, fathers and children were asked to participate in other home activities during the week. This included asking fathers and children to select from a variety of health challenges for themselves each week, including healthy eating, PA and TV challenges. Several main themes emerged, including children were excited about setting health challenges for their father. Family meals were already common and an important time to spend together as a family. Cooking healthily was viewed as a challenge, especially for traditional foods from Mexico and other Latin American countries. Some parents mentioned that many Hispanics lacked knowledge and desired to learn about how to cook healthy recipes at home.

*“To teach us to eat less fat, and to eat healthier foods, because the majority of Hispanic families, anything that is with oil, or fried stuff as they call it, is the tastiest but it’s the most damaging.”* (Father, interview, Spanish)

Fathers and children will be taught to use goal setting in the program. The main theme to emerge regarding goal setting was an appreciation for using individual goal setting to encourage behavior change for fathers and children.

*Start setting goals for how many healthy meals you eat a week until you don’t look at fast food, you don’t think about fast food*” (Father, interview, English)

e. *Ways to engage and inform mothers of the program*: If they were not invited to all the program sessions, most of the mothers preferred to receive program information through videos sent via Facebook. A variety of reasons emerged: there is no need to read it; it is a practical way to receive information; it is what people use most now-a-days; it is easier to access technology; and it is an easier way to learn for people who are more visual.

*“It’s very dynamic, very interactive. It shows images, drawings, which is what draws their attention. You can motivate them in that way as well, as mothers, in what they’re going to be involved with. In videos, they can understand it more. (*Mother, interview, Spanish).

Most mothers stated they are Facebook users, however not all use it regularly. They commented Facebook can send them notifications when tagged to a post in the group and they can comment on the posts. They liked the idea of learning about other mothers’ experiences through a Facebook group.

“*More than anything, if I were to be in a group like that, I would like to see their experiences, read every single thing they say, see their thoughts that in the long run help our children.*” (Mother, interview, Spanish)

### EVM dimension: content

Familism and gender roles emerged as the primary cultural content themes across the study activities, while spirituality and collectivism were minor cultural themes that emerged.

*Familism,* or the solidarity and strong attachment to the nuclear or extended family [26], was important to both fathers and mothers. Subthemes that emerged for *familism* included a strong identification and attachment to the nuclear family which was present when participants mentioned activities that the family enjoyed doing together in their free time; the importance of taking into consideration all members of the family; and fostering communication among family members. As previously mentioned, *familism* ties back to the desire to have mothers included more in the program to target the whole family during the program.

*Gender Roles persist in many Hispanic families*: Several subthemes emerged regarding gender roles. These included concern that Hispanic men’s sense of *machismo*, or masculine pride and responsibility as provider for the family, may prevent Hispanic men from wanting to participate.

“*… there is a lot in our culture about that, that the father doesn’t get involved in anything for the children, the mother is there, everything, the school, the doctor, everything. … but that is already in our culture, in our— that is how we were educated.”* (Mother, interview, Spanish)

A few thought that some men may interact differently with their sons and daughters in the program. Specifically, some Hispanic fathers may not want to do some of the physical activities with their daughters or be as expressive and warm with their sons.

***“****Well, my husband, he wrestles with my girls. But, we always say we are different. A lot of Hispanics I see like are “No, no. She’s a girl.“ Like, uh-uh. You don’t do that.” (Mother, focus group, English)*

The program could help change fathers’ belief that their main role is to be the breadwinner of the family, and allow him to spend more time and help care for his children.

“H*e [*husband*] thinks that men are supposed to work, but he is mistaken, they should also take care about the children stuff. It would be difficult maybe the first two times he goes [*to the program*], but I think he would like it*.” (Mother, Interview, Spanish)

Hispanic men recognized a generational change of attitudes among Hispanics fathers over their role in the family. Two primary subthemes emerged regarding generational change: older generations of Hispanic fathers may think this program is a waste of time, and younger generation of Hispanic fathers may have more equal views on playing with their daughters versus sons.

“… *I know a lot of, you know, Hispanos, like the dads are more … reluctant to just participate in something like that … As far as like my dad, … he’d probably be like you know this is a waste of time*. [However], *people in general … have like totally different attitude from … our parents or us being parents*.” (Father, interview, English)

***“****I think that for now-a-day guys, no. Times have changed.” Followed by … “Yes, I think what (Father participant) said is really good, and I was just going to tell him, that as you mentioned, it’s cultural and generational. So it wouldn’t be a problem if we include this within this program (referring to rough and tumble play with daughters).” (Father followed by Mother, focus group, Spanish)*

The competitive side of *machismo* would make fathers want to participate in the PA and health challenges in the program.

*“It’s in my father, I saw that a lot in him and I see that a lot with the guys that I work with. I mean, I’m going to lift -I don’t know- this concrete better than you. There’s always some kind of competition or some kind of machismo.”* (Father, focus group, English)

Some mothers were concerned their spouse may not be able to handle the children during the program without her help, reinforcing the cultural values of machismo and *marianismo*. *Marianismo* is the traditional gender role for mothers including to care for the children, while fathers have limited involvement in children’s day-to-day care.

4.c/d: *Spirituality and Collectivism*: The value of *spirituality* in Hispanic communities emerged as a minor theme. Some thought future versions of the program could be promoted in churches. *Collectivism* also emerged as a minor cultural value in ways to influence more fathers in the Hispanic community to participate. Having fathers feel like they belonged to a group with other members similar to themselves that had a sense of cohesion was viewed favorably.

### EVM dimension: persons

Two main themes emerged regarding the participant-facilitator relationship in a program. Participants preferred facilitators to be friendly, polite, flexible, sincere, and not pushy. This embodies the cultural concept of *Simpatia*, or geniality during interactions [26]. Fathers did not want to feel lectured and said program delivery must be engaging. One participant suggested the program have an “*orientation that is motivating*” (Father, interview, Spanish) to help establish a commitment from participants so they keep coming to all the sessions.

### EVM dimension: metaphors

EVM posits program material should engage the target population and align with the group’s cultural understanding of symbols and concepts that could affect recruitment and engagement in the program. A good example is the program’s name and logo. Several program names and logos for the culturally adapted HDHK program were tested with the parent panel. ‘Papás Saludables, Niños Saludables’ (Healthy Dads Healthy Kids) and ‘Que Padre, Tan Padre’ (What a cool Dad) were the favorites, but slightly more fathers preferred Papás Saludables, Niños Saludables. Several of the logos presented to the parent panel had silhouettes of a man and two children running, similar to the original logo for HDHK. One father raised concern over these and pointed out it reminded him of the signs at the US-Mexico border warning of families crossing the border on foot.

### EVM dimension: language

Three main themes emerged regarding parents’ and children’s thoughts and preferences for language and word choices.

*Participants self identified as Hispanic*: Participants preferred the term ‘Hispanic’ when describing their own ethnicity or cultural background. The main reason mentioned was the association with the Spanish language, whereas ‘Latino’ included other languages and non-Spanish speaking countries. No participants expressed they would feel offended by either of the two terms or other common terms to describe their ethnic group.

*Parents expressed appreciation* that the program would be delivered in Spanish or English, and thought it was important that all the material was available in both languages.

*There is a generational difference in language* preference between children and parents: The majority of children stated they preferred the program be in English for the children. Subthemes regarding their preference toward English found that many children were weary of voicing their preference to their parents due to their family/parent preference for Spanish, and parents had a preference for Spanish due to difficulty in communicating with family elders (grandparents, etc.) in English.

*“Well, my mom, my abuelita, want us to learn actually Spanish so we can talk to them so they won’t be that much confused because we’re talking in English”* (Boy, focus group, English)

### EVM dimension: context

Several context themes emerged for participation or engagement in the program, with several being barriers.

*Barriers for Hispanic fathers to participate in the program:* The most prominent subtheme to emerge was Hispanic fathers’ lack of time due to working long hours. Fathers’ lack of energy due to working physically demanding jobs and long hours, and lack of interest and motivation were other subthemes for barriers to participate. Transportation issues to get to the program venue was another barrier raised. Subthemes included families not having a car, poor public transportation in the area, or not having a driver license or other documents.

*Concern over children playing outdoors*: Neighborhood safety concerns and hot, humid weather for many months of the year emerged as barriers for children playing outdoors.

*Barriers to eating healthy*: Cost of healthy food, eating habits, and lack of time arose as barriers to eating healthier.

*Beliefs regarding food assistance programs*: Several subthemes emerged. Hispanic families who use food assistance programs needed guidance about how to prepare healthy meals with the assistance they receive. Immigration status prevented some from accessing food assistance programs. A difference in opinions emerged on whether HDHK should provide information about food assistance programs. Most participants liked the idea of adding information to the program about how to access food banks. Other participants thought it was a good idea to include information about Food Banks, but suggested to keep it confidential or optional because some may not have the official ID’s they believed were required to qualify.

“*Yes, food banks are very good, because some people don’t even know about food banks or think that they need certain things to go to a food bank, but in reality you just go get in line and get the food, but that would be a very good thing.”* (Mother, interview, English)

A few participants disliked the idea of including information about food banks because they believed it not helpful or even insulting.

“*… I think some would take it as an insult; a lot of people would be why are you giving me this you know. So I guess more of a pride thing.”* (Father, interview, English)

*Immigration status*: The immigration status of a few parents emerged as a barrier. Some reported an inability to get a driver’s license which caused them to have transportation problems. Additionally some voiced an inability to participate in some food assistance programs and fear to leave home and access public areas due to immigration status.

“*The fear that— the way the country is, I believe families prefer to stay home, even though a few years back you could simply take your children to kick the ball to a park, nowadays you cannot have that luxury*.” (Father, interview, Spanish)

#### Resulting program adaptations

A summary of the adaptations of the program that resulted from this formative work can be found in Table 4. Here we summarize the key adaptations of the HDHK program for the planned feasibility trial.

**Table 4:** Cultural Adaptation of Healthy Dads, Healthy Kids for Hispanic families

Culturally Centering Elements	Components retained from original program	Cultural adaptations to HDHK for Hispanic families
**goals**	• The original goals from HDHK o Teach fathers how their attitudes towards eating and physical activity influence the whole family o Encourage fathers to be healthy, positive role models for their family o Teach fathers effective ways to encourage healthy behaviors in their children o Assist to prevent or manage obesity in children o Help fathers achieve a healthy weight o Improve the relationship(s) between fathers and their child (ren) through physical activity and healthy eating	• Greater emphasis on child health and family health for goals of program • Greater emphasis on screen reduction and parenting based on desired goals from formative work • Added new message for reducing screen time for fathers and children in the culturally adapted program: switch off “por mi familia” (for my family)
**concepts**	• The focus on fathers with a gender-tailored program • Fathers as role models • Focus on fathers’ attitudes on healthy eating and physical activity influences his whole family • Promotion of authoritative parenting	• Reciprocal reinforcement was retained, but adapted with the addition of *Respeto* when teaching children to be their fathers’ health coach • Add a more explicit discussion with fathers on the importance of letting their kid be their health coach to allow the child to learn healthy lifestyle behaviors. • Greater focus on the child coaching the whole family toward healthy eating and physical activity
**methods**	• The 90 min structured weekly sessions (welcome/check in, separate practical components for father and children, combined fun PA component for fathers and children) • The three components of the PA portion; Rough and Tumble Play, Sports Skills (Fundamental Movement Skills) and Fun Fitness. • Provided adapted handbooks to Fathers, Children and Mothers • Children were asked to select and complete home tasks every week and have their father sign off • Fathers were asked to set monthly SMART goals • Fathers were asked to select and complete weekly home tasks • Fathers advised to decrease dietary intake by 500 cal/day if they desire healthy weight loss • Fathers and children were provided with pedometers and asked to track their steps. Encouraged to set challenges for each other to increase steps. • Fathers were asked to weigh-in every week and track their weight • Fathers and mothers taught the principle of ‘parent provide, children decide’ to encourage healthful intake at meals.	• Lowered the reading level of all material, including eliminating complex statistics and graphs • Health statistics were changed to reflect data on Hispanics in the US • All physical activity, eating and screen media recommendations were changed to reflect the most recent US recommendations. • The concept of Respto (respect) was introduced to the children when talking about Rough and Tumble Play • Slides were changed to include more images to depict concepts rather than text • Changed photos throughout program material to reflect Hispanic families, fathers, and children • Food and dietary content was changed to reflect common Hispanic foods • More examples of easy shifts in dietary intake to reduce caloric intake were provided • Added images of what 500 cal look like (5 flour tortillas, large fries, 2 pieces of pan dulce, etc.) • Greater discussion on decreasing added sugars to daily intake with examples provided • Added a booster session at week 6 • Sessions were reordered, so that reducing screen time was moved to earlier in the program to address parents’ desire to make that a more prominent goal of the program • Encouraged even more group interaction and discussion to minimize didactic portion • Simplified process for setting SMART goals • Simplified the process for fathers to calculate their personal energy requirements for weight loss and provided each father with a customized card (rather than having them calculate it themselves) • Selected dietary intake self-monitoring app available in Spanish and English that had the most complete set of common Hispanic foods listed • Provided families with a USDA sponsored Cookbook available in Spanish and English on healthy Hispanic recipes: (Flavors of my kitchen; California Department of Public Health. USDA SNAP, 2015) • Engage mothers in a different way o Weekly videos via Facebook or text o Secret Facebook group o Text reminders
**content**	• Fathers were encouraged to participate with their daughters and sons (*gender roles*).	• Added discussions for fathers about differences between their own attitudes about what it means to be a father compared to that of their own father’s generation (e.g. *generational shift*). • Added discussions of raising children in a different culture than their own. • Promoted importance of health of whole family (*familism*). • Fathers were given specific pointers on how to start meaning conversations with their child and how to actively listen to their child to help fathers feel like they can get more involved with their children (*gender roles*). • Promoted the group were cohesive and encouraged them that other Hispanic fathers also wanted to get closer to their children and be good role models for their children (*collectivism*).
**persons**	• Facilitators should be credible, relatable, and likable	• Facilitators should be bilingual and preferably from the same or similar culture as the families • Facilitators to demonstrate *simpatia*, while being fun and energetic
**metaphors**	• Dad jokes, in Spanish	• New program logo
**language**		• Translated into Spanish, focus on content not direct translations • Revised English to reflect America-English, rather than Australian-English • Used the term Hispanic when referring to the target population based on their preference • The games on the play cards were called by the names parents remembered from their childhood or substituted for games they recalled that practiced the same skill.
**context**	• The program will continue to emphasize quality of time, rather than quantity of time fathers are able to spend with their children.	• The program will be delivered on the weekend, when Hispanic fathers have more time. • The benefit of leisure time PA to reduce stress in a way PA from physical demanding jobs cannot has been added. • Added information on eating on a budget • Added optional resources on food assistance programs in the local area • Identified games fathers and children can play indoors on the game cards • Transportation will be provided to families who report lack of transportation as a barrier to program attendance

The most important core component of HDHK was the focus on fathers, which defines the unique element of the intervention. However, one significant theme that emerged across the study activities was the importance of *familism* among Hispanic families that needed to be considered. *Familism*, or the strong attachment and reverence for the nuclear family was apparent when discussing what the family did together and how they conceptualize health and happiness. Throughout the interactions several participants expressed a desire to include mothers in all program sessions to promote this cultural value, but this was not universally expressed among all participants. Others preferred the focus on fathers. The research team was concerned that including mothers in all components would change the program to a parenting or family intervention and impact father engagement and attendance. Based on the experience of previous studies [11] this would likely result in few fathers participating, maybe because they abdicate the participation to mothers or because they do not feel comfortable taking on new roles in the presence of women. Ultimately, the concern was that engaging mothers in all aspects of the program would remove the focus on fathers and thereby negatively affect a core component of the program. Instead, the program was kept as a gender-tailored program for fathers and their children, but was augmented with additional ways to engage mothers. This included developing short videos for mothers to deliver the same content to them weekly and creating a closed Facebook group for mothers to engage with the program facilitator and other mothers, both ideas which were well-received by mothers on the Family Panel.

Another core component of HDHK was reciprocal reinforcement, over which some Hispanic parents and children reported concern. The panel had positive reactions to fathers becoming role models for their children for healthful eating and PA. However, they suggested that some Hispanic fathers would not appreciate or listen to their child if she/he were to tell father to eat healthy and be active. Since reciprocal reinforcement was pre-identified as a core component of HDHK, we did not remove reciprocal reinforcement from the culturally adapted program. Instead, how the concept of children as supporters for their fathers will be introduced and operationalized to children and their fathers was adapted. First, the concept of *respeto* (respect) for their father will be taught to children when supporting their fathers to be healthy role models. The desire is to link back to another cultural value, so when the child encourages or helps her/his father to eat healthy and be more active it will be better received by the fathers. The timing of making the suggestion, the words chosen (helpful encouragement instead of demands), and the opportunity to make changes together will be discussed with the children. A greater emphasis on engaging the whole family in behavior change, rather than just the father, will be promoted throughout the sessions to also link it back to *familism*. In the culturally adapted program, fathers will also be told in the first session that their child will encourage them to change their eating and activity behaviors in order for the child to adopt more healthy behaviors herself/himself.

Gender roles for men and women in Hispanic families surfaced as a significant theme that needed to be acknowledged and addressed in the culturally adapted version of HDHK. In the original Australian version, the program was gender tailored for men, suggesting that gender roles exist in many cultures and families. However, gender roles may be more prominent and significant in Hispanic families than in many others. In the original program, the important and different role that fathers play in their children’s life compared to mothers was emphasized. This was retained with additional discussions about the generational change that has occurred for many Hispanic fathers as brought out by the Family Panel. In addition, the program discussion that PA, sports, and rough and tumble play benefit both girls and boys was enhanced. The new Papás Saludables, Niños Saludables logo included a father with a girl and boy playing soccer, with the girl being actively engaged in kicking the ball to illustrate the inclusion of girls in the PA portions of the program. This also addressed an important metaphor issue identified by the Family Panel (border crossing warning sign image linked to initial logo tested).

Many of the barriers identified by the parents of the Family Panel related to their context and the methods for how the program’s goals will be achieved were adapted. The adapted program acknowledges the amount of time many fathers spend working within the Hispanic community. They will be encouraged to continue to attend the program, even if they miss sessions due to work. The physically demanding jobs often held by Hispanic men will also be recognized, but the benefit from being active during recreational time compared to at work [27, 28] will be highlighted. All of the material in the program, including the handbooks, instructional sheets, game cards, and slides were simplified with reduced reading levels, removal of complex graphs and instructions, and use of images to relay messages instead of text when possible. A booster session will be added at week six, to review the content taught to date, home activities, and the monitoring fathers and children will be asked to do. The goal was to allow time to review any content that the fathers did not understand or require additional instruction after a first attempt at the activity. To address the barriers to eating healthy, a United States Department of Agriculture (USDA) developed cookbook in Spanish or English of how to make healthy versions of common Hispanic meals will be provided to all families. Many examples of how to reduce calories with simple shifts in eating practices will be provided using common foods consumed by Hispanic families in this region, and information of how to eat on a budget and food assistance programs will be provided. The adapted game cards include and identify games that could be played indoors for families who live in unsafe neighborhoods or for when it is too hot to play outdoors. Transportation barriers will be assessed, and transportation to the program may be provided on a case-by-case basis (due to funding limitations).

Lastly, adaptations were made to address other cultural components that emerged under concepts, methods and content. Based on the theme that some fathers have a desire (but often do not know how) to bond with their children, advice and practice on conversation starters with children will be provided. Given that parents’ immigration status was raised, a discussion was added about raising children in a different culture than where you grew up, with an emphasis on how to set rules for your child that are reasonable within the new culture. In addition, all the program material was adapted for US dietary guidelines [29], PA recommendations [30], and screen media guidelines [31]. Images and examples were changed to reflect Hispanic families, common Hispanic foods, and preferred PA and sports.

## Discussion

This paper describes a systematic approach to culturally adapt the HDHK program for a new population and context from those targeted by the original HDHK program. As recommended by others [32, 33], stakeholder input was critical in informing the adaptation. Stakeholders for this project included representatives of the target population (Family Panel) with similar inclusion and exclusion criteria as for the planned feasibility trial. Additionally, the developer of HDHK (PM), and researchers with expertise in Hispanic fathers, health promotion among Hispanic families, general parenting within Hispanic families, and cultural adaption of evidence based parenting programs (Expert Panel) were important stakeholders. Iterative input from the Family and Expert Panels guided the decisions of the cultural adaptation of the HDHK program for Hispanic families. The focus on fathers was maintained in the resulting Papás Saludables, Niños Saludables program, but mothers were integrated more to address the important cultural value of familism within the Hispanic community. Additionally, the benefit of nutrition, PA and screen media behavior changes for the *whole* family was emphasized to a greater extent to further operationalize familism. Core components of HDHK were identified a priori and retained as recommended by guidelines for program adaptation [32, 33]. However, the core component of reciprocal reinforcement was adapted to address cultural concerns of children telling their father what to do. Gender roles for fathers and mothers were acknowledged, but additional discussions were added on the changing role of Hispanic fathers with their children, both daughters and sons. Father-child bonding over fun active games and health promotion was retained and emphasized. Barriers for Hispanic fathers’ behavior change and program engagement were addressed, such as delivering the program on the weekend due to fathers’ lack of time during weekdays. The lower education level of many of the low-income, Hispanic men and resulting lower literacy level was addressed by multiple modifications of the program materials and adding a booster session to review and check in on learning, midway through the program. Several adaptations were made to the program to make it more relevant and interesting for Hispanic families, such as referring to games that Hispanic fathers remembered from their childhood and giving out a cookbook with healthy versions of familiar Hispanic dishes.

Adapting existing evidence-based interventions for different contexts has received greater attention in recent years. Since the Papás Saludables, Niños Saludables project was started, two groups have provided guidance on best practices for systematically adapting existing health promotion programs for new contexts [32, 33]. Both guidelines emphasize the critical role understanding contexts has on delivering programs that are appropriate, implementable, effective and sustainable for new target groups. The cultural adaptation of HDHK into the Papás Saludables, Niños Saludables program addressed the context of low-income Hispanic families in the US and included both surface structural changes (e.g. changing images, foods and games) to the material and deep structural changes that addressed cultural values of the target population [34]. By addressing both, the goal was to improve the acceptability, relevance and ultimately the impact on participating Hispanic families. Systematic cultural adaptation of programs may be particularly important when adapting a healthy lifestyle program that targets Hispanic fathers and their children who experience health disparities. Adequately considering the context of new target populations is critical to avoid widening health inequalities [33]. For example, findings from the Family Panel identified that low-income Hispanic families experience intense contextual stressors, such as immigration-related challenges, strenuous working conditions, and barriers to accessing food resources in their community. These stressors, along with other significant barriers to access health services and distrust of health care providers due to instances of discrimination [35, 36] highlight the need to provide culturally relevant programs for Hispanic families.

Hispanic families have been targeted in a number of child obesity prevention trials [37] and one ongoing study targets Hispanic men with a culturally-adapted weight loss intervention [38]. However, Hispanic fathers and their children have never been the specific target of a child obesity prevention program. Hispanic fathers are an important, understudied, and novel target for weight management and child obesity prevention. Utilizing a comprehensive framework, such as the EVM, to inform the cultural adaptation of HDHK for Hispanic fathers with qualitative input from both an Expert Panel and a Family Panel is innovative for family-based obesity programs. The EVM framework allowed for a thorough and systematic approach in identifying program concepts and content that required adaptation for a new target group, while retaining the core components of an established program. A few other groups have culturally adapted existing family-based lifestyle or weight management programs [39, 40] or designed such programs specifically for Hispanic families with formative studies informing the program [41–43] to enhance the relevance for Hispanics families. However, to our knowledge, utilizing an established, comprehensive framework to inform the adaptation of family based obesity interventions to help ensure external validity of evidence-based programs when targeting new populations has not previously been done. The obesity prevention, nutrition, and PA fields should consider applying systematic approaches [32, 33] when adapting evidence-based programs to any new target population.

Another research group has recently adapted HDHK for another context, socioeconomically disadvantaged, ethnically diverse fathers and children in the United Kingdom. Similar to this study, they engaged representatives of the target population in interviews and focus groups, in addition to formative data from other on-going studies to inform their adaptation of HDHK. The resulting adaptations included changes in the recruitment and follow-up procedures, program delivery and program content, primarily at a surface structural level [44]. After two feasibility trials of the adapted HDHK-UK program, the researchers concluded that despite the program being delivered with high fidelity and well received among participants, there were significant challenges in recruiting overweight or obese men and low follow up rates, resulting in the adapted program not being evaluated in an efficacy trial. In contrast, the systematic approach of adapting HDHK into Papás Saludables, Niños Saludables was guided by a framework and involved both surface and deep structural adaptations to the HDHK program [34]. Papás Saludables, Niños Saludables will next be tested for feasibility in the US and outcomes can be compared to the UK experience.

While it is important to obtain input from the target population when culturally adapting a lifestyle program, the formative studies described here had limitations that should be acknowledged. The participants were asked to provide input and feedback about a program in which they had never participated. Instead, they were provided overviews of the program in the form of short videos, PowerPoint presentations, and example material, but truly experiencing a program would provide a different vantage point. It is therefore equally important to conduct exit interviews of participants who will take part in the planned feasibility trial of the culturally adapted program. Most participants did not participate in every research activity. The majority took part in three or less activities. This may be a limitation in that the more exposure to the program material and content, the greater understanding the participant should have of the program. Alternatively, a rolling recruitment to keep our family panel intact provided input from a broader range of individuals who are similar to the planned target group of the future feasibility trial.

## Conclusion

The use of a conceptual framework, such as EVM, when culturally adapting an intervention ensures the adapted version supports external validity and increases the probability that the new target group will be interested and engaged in the program. This qualitative formative study informed the cultural adaptation of HDHK for Hispanic families, including decreasing the literacy level, integrating cultural values and addressing barriers for participation and engagement, without losing the core components of the original program. The next step will be to test the feasibility of the culturally adapted HDHK program among Hispanic families and assess whether it is appropriate to then move on to an efficacy trail of the culturally adapted Papás Saludables, Niños Saludables program.

## Data Availability

The de-identified data are available from TMO upon reasonable requests.
